# Retraction Note to: The effect of endometrial injury on first cycle IVF/ICSI outcome: A randomized controlled trial

**DOI:** 10.18502/ijrm.v20i12.12571

**Published:** 2023-01-09

**Authors:** Ahmad Mahran, Mahmoud Ibrahim, Haitham Bahaa

Published online: 29 March 2016

Retraction to: Int J Reprod BioMed (2016) 14: 193-198

doi.org/10.29252/ijrm.14.3.193

This article has been retracted at the request of the IJRM editorial board, based on the results of an investigation which found serious flaws in the study results.

The online version of the original article can be found at http://ijrm.ir/article-1-733-en.html

Ahmad Mahran, Mahmoud Ibrahim, Haitham Bahaa.

Department of Obstetrics and Gynecology, Faculty of Medicine, Minia University, Minia, Egypt,

**Corresponding Author:**

Ahmad Mahran; Department of Obstetrics and Gynecology, Faculty of Medicine, Minia University, Minia, Egypt.

Mahran A email: ezzeldin_ahmad@yahoo.com

Ibrahim M

E-mail: hosnimahmoud60@yahoo.com

Bahaa H

E-mail: haitham_bahaa@yahoo.com

